# Quality of Life in Patients With Inflammatory Bowel Disease: Importance of Psychological Symptoms

**DOI:** 10.7759/cureus.28502

**Published:** 2022-08-28

**Authors:** Maria-Andriani Mitropoulou, Evangelos C Fradelos, Ka Y Lee, Foteini Malli, Konstantinos Tsaras, Nikolaos G Christodoulou, Ioanna V Papathanasiou

**Affiliations:** 1 Nursing, University of Thessaly, Larissa, GRC; 2 Nursing, University of Thessally, Larissa, GRC; 3 Health Sciences, Mid Sweden University, Östersund, SWE; 4 Psychiatry, University of Thessaly, Larissa, GRC

**Keywords:** ulcerative colitis (uc), crohn’s disease (cd), inflammatory bowel disease, psychological symptoms, quality of life (qol)

## Abstract

Background

Depressive, anxiety, and stress symptoms are prevalent in patients with inflammatory bowel disease (IBD) and may negatively influence the disease course and quality of life. The study aimed to assess the relationship between psychological factors and patients’ quality of life (QoL).

Materials and methods

A cross-sectional study with the use of a structured questionnaire among 38 patients with Crohn’s disease and 30 with ulcerative colitis was conducted. The inflammatory bowel disease questionnaire (IBDQ-32) was used to evaluate the patients’ quality of life, and the Depression, Anxiety, Stress Scale (DASS21) was used to evaluate these psychological factors. Pearson descriptive statistics and multiple regression analyses were performed.

Results

According to the findings of the multiple regression analysis, depressive, anxiety, and stress symptoms were negatively associated with quality of life. Participants with higher scores of anxiety had inferior QoL in intestinal symptoms (p=0.013) and in systemic symptoms (p=0.013), with higher scores of depression had inferior QoL in emotional function (p<0.001), and higher scores of stress had inferior QoL in the domain of social support (p=0.002). Psychological symptoms of emotional disorders appear to be associated with lower quality of life in IBD patients.

Conclusion

This study examined levels of depression, anxiety, and stress in Greek patients with IBD, which were associated with lower levels of their quality of life. Interventions to improve QoL in patients with IBD should consider the effect of psychological symptoms.

## Introduction

Inflammatory bowel disease (IBD), including Crohn's disease and ulcerative colitis, is a chronic inflammatory disorder of the gastrointestinal tract and is often accompanied by embarrassing bowel and systemic symptoms [1]. Recent studies have reported an increased risk for anxiety or depression in both children and adults with IBD [2]. Psychological factors have a special role in this disease as they can trigger the clinical appearance of the symptoms or modify IBD symptoms [3]. The bidirectional relationship between IBD and psychological problems can be explained in terms of the "brain-gut" axis, meaning that the presence of anxiety and depressive symptoms or disorders can increase intestinal inflammation and may contribute to disease relapse, and, conversely, intestinal inflammation can negatively influence mood [4].

Quality of life (QoL) can be defined in many ways, making its measurement and incorporation into scientific study challenging. QoL is defined by the World Health Organization as "an individual's perception of their position in life in the context of the culture and value systems in which they live and in relation to their goals, expectations, standards, and concerns" [5]. The symptoms of IBD have a negative impact on patients' well-being and quality of life because dysfunctions usually affect daily activities, performance in school, ability to work, and social life [6]. Mental health is an important part of caring for these patients but is often overlooked.

Literature Review

IBD is a chronic inflammatory disease of the gastrointestinal tract and is divided into Crohn's disease and ulcerative colitis. It occurs in genetically susceptible individuals after an exaggerated immune response to a normal stimulus such as intestinal flora [1]. The relapsing and remitting course and chronic nature of IBD mean that patients frequently deal with uncomfortable symptoms and side effects of medications used to treat these symptoms, which can lead to lower quality of life (QoL) [7]. Several factors have been identified that influence QoL in IBD, including disease activity, psychological status, fatigue, illness perceptions, coping strategies, and social support. Health-related quality of life is a multidimensional measure that reflects the impact of IBD on an individual's physical, psychological and social functioning [8].

According to the results of a systematic review, individuals with IBD have higher rates of both anxiety and depression symptoms compared with healthy individuals without IBD [9]. There is an increased incidence of psychiatric disorders, mainly depressive and anxiety disorders, after the diagnosis of IBD [9, 10]. Previous research has highlighted that stress and depression are higher during active illness compared to inactive illness and quality of life scores appear lower, particularly during an event [11]. A meta-analysis referring to the evaluation of patients living with IBD showed the adverse effect of the disease on QoL [12]. The symptoms of IBD disrupt interpersonal relationships, daily activities, social participation, and mental well-being, affecting the QoL of both patients and their caregivers [12].

Overall, the psychological difficulties, such as depression, anxiety, stress, and social isolation, experienced by IBD patients seem to be associated with lower QoL. Studying this association may improve care for patients with IBD. The present study aimed to assess the relationship between QoL and psychological symptoms.

## Materials and methods

Study Design

A cross-sectional study using a structured questionnaire (IBDQ-32) was conducted. The convenience sample consisted of IBD patients in Greece. The questionnaire was offered online to members of the official association of people with Crohn's disease and ulcerative colitis in Greece - Hellenic Society of Crohn's Disease and Ulcerative Colitis Patients (HELLESCC). Data collection lasted from March 2021 to July 2021. The online method of administration was necessary due to the Covid-19 pandemic and in order for the sample to represent as many different regions of Greece as possible. The sample consisted of 68 patients that agreed to participate in the current study and successfully answered all the questions of the questionnaire. Of the 68 patients, 38 (55.88%) are diagnosed with Crohn's disease, and 30 (44.12%) have ulcerative colitis. The inclusion criteria were the following: consent to participate in the study, 18 or more years of age, ability to communicate in the Greek language, and an official diagnosis of IBD.

Ethics

This study was conducted in compliance with the ethical standards. The study protocol was approved by the institutional review board (IRB) of the official association of people with Crohn's Disease and Ulcerative Colitis in Greece, HELLESCC (IRB approval No:021/26.03.2021). The study adheres to the Declaration of Helsinki, and informed consent was obtained from participants. In addition, licenses for the questionnaires were issued from the respective researchers.

Study Measures

The Inflammatory Bowel Disease Questionnaire 32 (IBDQ-32) and the Depression, Anxiety, and Stress Scale-21(DASS-21) were used in the current study. The Inflammatory Bowel Disease Questionnaire 32 (IBDQ-32) is one of the most commonly used measures to assess IBD patients' QoL. IBDQ-32 is a 32-item questionnaire that includes four aspects of the patient's life: intestinal symptoms (10 items), systemic symptoms (five items), social (12 items), and emotional domains (five items). The answers are on the Likert scale from 1 (worst case) to 7 (best case), which indicates how much the statement applied to them during the last two weeks, with lower scores reflecting worse QoL. The questionnaire was translated and validated in Greek by Pallis et al., and Cronbach's alpha (= 0.78) has been tested for validity and reliability [13]. The Depression, Anxiety and Stress Scale-21 (DASS-21) is a set of three self-report scales designed to measure the emotional states of depression, anxiety, and stress. Each of the three DASS-21 scales contains seven items, divided into subscales with similar content. Participants answer on a four-point Likert scale (0 = did not apply to me at all, 3 = it was too much for me, or most of the time) which indicates how much the statement applied to them during the previous week. Scores for depression, anxiety, and stress are calculated by summing the scores for the relevant items. The questionnaire was translated and validated for the Greek population by Lyrakos et al., and Cronbach's alpha of the scale was α=0,965, which suggests that the Greek translation of the DASS-21 is both reliable and valid [14]. A series of demographic and clinical characteristics such as age, gender, and marital status were also recorded.

Statistical Analysis

Mean, standard deviation (SD), median, and interquartile range were used to describe the quantitative variables. Absolute (N) and relative (%) frequencies were used to describe the categorical variables. The Student's t-test or the non-parametric Mann-Whitney criterion was used to compare quantitative variables between two groups. The parametric analysis of dispersion analysis (ANOVA) or the non-parametric Kruskal-Wallis criterion was used to compare quantitative variables between more than two groups. To reduce the type I error due to multiple comparisons, Bonferroni correction was used, according to which the significance level is 0.05/k (k = number of comparisons). To correlate the relationship between two quantitative variables, Pearson or Spearman correlation tests were used. The correlation is considered low when the correlation coefficient (r) ranges from 0.1 to 0.3, moderate when the correlation coefficient ranges from 0.31 to 0.5, and high when the coefficient is greater than 0.5. Linear regression analysis with stepwise integration was used to find independent factors related to DASS-21 and IBDQ, from which dependencies (b) and their standard errors (SE) were derived. The linear regression analysis, where necessary, was performed using logarithmic transformations. The significance levels are bilateral, and the statistical significance was set at 0.05. The statistical program SPSS version 22.0 (IBM Inc., Armonk, New York) was used for the analysis.

## Results

The study population included 68 patients whose demographic and clinical characteristics are presented in Table 1.

**Table 1:** Demographic and clinical characteristics of the participants (n=68)

Demographic characteristics			Ν	%
Sex	Male		23	33.8
	Female		45	66.2
Age in years, mean (SD)			39.3 (13.5)	
Educational attainment	Junior high school/high school		17	25
	University student		11	16.2
	Graduate		28	41.2
	Postgraduate		9	13.2
	PhD		3	4.4
Family status	Married/partnered		34	50
	Alone		28	41.2
	Divorced		6	8.8
Children	No		34	50
	Yes		34	50
Employment status	Unemployed		17	25
	Employed		46	67.6
	Semi employed		1	1.5
	Pensioners		4	5.9
Live with	Alone		16	23.5
	Friends		2	2.9
	Family		49	72.1
	Caregivers		1	1.5
Living area	Urban area		52	76.5
	Semi-urban area		11	16.2
	Rural area		5	7.4
Clinical characteristics			Ν	%
Suffer from		Crohn’s disease	38	55.9
		Ulcerative colitis	30	44.1
Age of first diagnosis of IBD in years, mean (SD)			28.5 (13.3)	
Family members who suffer from IBD		No	54	79.4
		Yes	14	20.6
The disease is active in the current period		No	47	69.1
		Yes	21	30.9
Have you ever been hospitalized for this disease?		No	23	34.3
		Yes	44	65.7
How many times in total have you been hospitalized because of this disease? SD, median			2,6 (5,5)	1 (0-3)
Have you had any surgery related to this disease		No	59	86.8
		Yes	9	13.2
Do you suffer from another serious disease?		No	58	85.3
		Yes	10	14.7
If yes, choose		Anemia	1	1.5
		Asthma	1	1.5
		Hard of hearing	1	1.5
		Idiopathic thrombocytosis	1	1.5
		Osteoporosis	1	1.5
		Diabetes	1	1.5
		Multiple sclerosis	1	1.5
		Systemic lupus erythematosus	1	1.5
		Hypothyroidism, vitiligo, psoriasis, psoriatic arthritis	1	1.5
		Hashimoto's disease	1	1.5

All participants were Greek. Of them, 66.2% were women, and the mean age was 39.3 years (SD = 13.5 years). Concerning the clinical characteristics, a total of 55.9% of participants suffered from Crohn's disease and 44.1% from ulcerative colitis. The mean age at the first diagnosis of IBD was 28.5 years (SD = 13.3 years), with 20.6% of participants having someone in their family who suffered from IBD and 30.9% having active disease at the time of the study. In addition, 65.7% of the participants had been hospitalized at least once due to the disease, 13.2% had undergone surgery related to the specific disease, while 14.7% had another significant disease.

The descriptive results for the scores on the dimensions of the scale of anxiety, depression, and stress are presented in Table 2.

**Table 2:** Patients' depression, anxiety and stress score (n=68)

	Minimum value	Maximum value	Mean (SD)
Depression score	0	42	14.3 (11.5)
Anxiety score	0	42	11.1 (10.1)
Stress score	0	42	18.5 (11.5)

The scores range from 0 to 42 points with a higher score indicating higher levels of anxiety, depression, and stress. The average score for depression was 14.3 points (SD = 11.5 points), for anxiety was 11.1 points (SD = 10.1 points), and for stress was 18.5 points (SD = 11.5 points).

The descriptive results for the scores in the Greek version of IBDQ are presented in Table 3.

**Table 3:** IBD patients' quality of life score (n=68) IBD - inflammatory bowel disease

	Minimum value	Maximum value	Mean (SD)	Median
Intestinal symptoms	24	70	55.2 (11.4)	58.5 (47.5-63)
Systemic symptoms	11	35	23.8 (5.9)	24 (20-28)
Emotional function	26	76	56.2 (13.7)	57.5 (45-68)
Social function	13	35	29.5 (6.2)	32 (25-35)

Higher scores ​​indicate an increased quality of life. The score for intestinal symptoms ranged from 24 to 70 points, with the average value being 55.2 points (SD = 11.4 points), and the score for systemic symptoms ranged from 11 up to 35 points, with the average value being 23.8 points (SD = 5.9 units). Also, the score for emotional function ranged from 26 to 76 points, with the average value being 56.2 points (SD = 13.7 points), and for social activity, it ranged from 13 to 35 points, with the average value being 29.5 units (SD = 6.2 units).

The multiple linear regressions were performed, where the IBDQ-32 dimensions of "intestinal symptoms", "systemic symptoms", "emotional function", and "social activity" were used as dependent variables, and the demographic-clinical data and the scores of anxiety, depression, and stress were used as independent variables. The analyses were performed by the bidirectional stepwise regression, which is a combination of the forward and backward methods, testing at each step for variables to be included or excluded and by the use of logarithmic transformations.

Anxiety symptoms were independently associated with intestinal and systemic symptoms, depressive symptoms with emotional function, and stress symptoms with social function, domains of quality of life in IBD patients. In addition, the activity of disease was independently associated with intestinal, systemic symptoms, and social function (Table 4).

**Table 4:** Multiple linear regression (stepwise) with dependent and independent variables Dependent variables: intestinal symptoms, systemic symptoms, emotional function, and social activity (from the IBDQ-32); independent variables: demographic-clinical data, scores of anxiety, depression, and stress. IBDQ-32 - Inflammatory Bowel Disease Questionnaire 32,

		β+	SE++	p-value
Intestinal symptoms of IBDQ-32 and anxiety				
Anxiety		-0.004	0.001	0.001
The disease is active in the current period	No			
	Yes	-0.057	0.024	0.019
Systemic symptoms of IBDQ-32 and anxiety				
Anxiety		-0.004	0.002	0.013
Live alone	No			
	Yes	0.058	0.029	0.045
Emotional symptoms of IBDQ-32 and depression				
Depression		-0.005	0.001	<0.001
The disease is active in the current period	No			
	Yes	-0.076	0.026	0.005
Social function of IBDQ-32 and stress				
Stress		-0.003	0.001	0.002
The disease is active the current period	No			
	Yes	-0.08	0.025	0.002

## Discussion

The aim of this study was to investigate the levels of depression, anxiety, stress, and quality of life of patients with inflammatory bowel disease (IBD) and to examine possible correlations between the aforementioned variables. Anxiety and depression are the most common psychological disorders in patients with IBD, and patients' self-reports of anxiety and depression are considered critical to the care of these patients [15, 16]. In addition, a population-based study reported that the prevalence of anxiety and depression during the lifetime of patients with IBD was 24.4-31.9% and 21.8-22.5%, respectively [17]. This study found patients with IBD reported higher levels of depression and stress compared to anxiety.

IBD has a significant impact on quality of life. A French cohort showed that half of IBD patients report poor quality of life and severe fatigue [18]. The symptoms of the disease disrupt interpersonal relationships, daily activities, social participation, and mental well-being, affecting the quality of life of both sufferers and their caregivers [19]. For these reasons, the assessment of the quality of life in patients with IBD is increasingly recognized by the medical and scientific community. Investigating the dimensions of patients' quality of life, the data showed that this Greek sample reports a good quality of life in terms of emotional function (56.2±13.7). These results are not very different from the results of a previous study that assessed health-related QoL of eighty-nine IBD patients in South-Western Greece and in which the score of emotional function was 67 for participants with Crohn's disease and 68 for patients with ulcerative colitis [20]

In this study, IBD patients with symptoms of anxiety were predisposed to have decreased quality of life in the domains of intestinal and systematic symptoms. The chronic nature and severity of symptoms cause stress and anxiety in patients, which were associated with lower quality of life [21]. Symptom-related concerns such as anxiety about losing bowel control, fatigue, fear of sexual dysfunction, social isolation, anxiety about not being able to function, fear of stigma, and feeling unclean are associated with levels of psychological distress [22]. In addition, results showed that the higher the participants' depression levels, the lower their quality of life was in terms of their emotional function. Studies describe a two-way relationship between psychological problems and inflammatory activity. A previous bad psychological status may contribute to IBD symptoms, and, in turn, inflammatory activity is associated with the development of psychological disorders [23]. Depression is associated with a reduced response to treatment and periods of flare-ups [24]. Therefore, it is important to simultaneously look after emotional and physical well-being in order to ensure a good clinical course of the disease. In a recent study in which 70 patients diagnosed with IBD participated, it was found that positive psychological factors, such as body acceptance and meaning in life, are significant predictors of QoL [25]. Furthermore, a study conducted on 172 patients with IBD found that the disease severity index that predicts disease severity in IBD patients was significantly higher in patients with symptoms of moderate-severe stress, depression, anxiety, and impaired QoL [26].

Also, increased self-reported levels of stress were associated with decreased quality of life in areas of social activity. Physical symptoms often have an inhibitory effect on a person's social activities, which is associated with increased stress. Similarly, the ability to work and participate in social activities is affected by high levels of psychological distress and leads to lifestyle changes and disturbances in social relationships and family life [22]. Studies show that the main factors that affect the quality of life of patients, both adults and children, are the presence of the symptoms, the severity of the disease, and emotional disorders [27, 28]. In this study, we found that participants with active disease had inferior QoL in intestinal symptoms, emotional function, and social support.

IBD patients' quality of life and their mental status are also receiving attention. Screening patients with IBD for mood disorders has recently been shown to provide mental health benefits [11]. The effects of an untreated mental illness can be devastating and can worsen the disease course of IBD [22]. So, it is important to timely recognize mental disorders in the IBD population.

Limitations

The current study contains several limitations, including a small sample size. Collecting data online could lead to sampling bias. In addition, this study relied on self-reported data regarding patients' diagnoses. Finally, this is a cross-sectional study in which no causal relationship can be tested. Therefore, future longitudinal studies including both adults and children are warranted.

## Conclusions

This study delineated the impact of symptoms of anxiety, depression, and stress on a Greek IBD population. Emotional difficulties experienced by patients affect the biological, psychological, and social areas of their lives. Our findings emphasize the importance of a holistic approach and treatment of chronic IBD diseases. These patients should have timely access to multidisciplinary care, including mental health professionals, to control and manage psychological distress.
